# Diversity of gastrointestinal helminths in Dall's sheep and the negative association of the abomasal nematode, *Marshallagia marshalli*, with fitness indicators

**DOI:** 10.1371/journal.pone.0192825

**Published:** 2018-03-14

**Authors:** O. Alejadro Aleuy, Kathreen Ruckstuhl, Eric P. Hoberg, Alasdair Veitch, Norman Simmons, Susan J. Kutz

**Affiliations:** 1 Department of Biological Sciences, University of Calgary, Calgary, Canada; 2 Museum of Southwestern Biology and Department of Biology, University of New Mexico, Alburquerque, NM, United States of America; 3 124 York Court, New Glasgow, Nova Scotia, Canada; 4 Producers of Diamond Willow, Pincher Creek, Alberta, Canada; 5 Department of Ecosystem and Public Health, Faculty of Veterinary Medicine, University of Calgary, Calgary, Canada; Universitat Autonoma de Barcelona, SPAIN

## Abstract

Gastrointestinal helminths can have a detrimental effect on the fitness of wild ungulates. Arctic and Subarctic ecosystems are ideal for the study of host-parasite interactions due to the comparatively simple ecological interactions and limited confounding factors. We used a unique dataset assembled in the early seventies to study the diversity of gastrointestinal helminths and their effect on fitness indicators of Dall’s sheep, *Ovis dalli dalli*, in the Mackenzie Mountains, Northwest Territories, Canada. Parasite diversity included nine species, among which the abomasal nematode *Marshallagia marshalli* occurred with the highest prevalence and infection intensity. The intensity of *M*. *marshalli* increased with age and was negatively associated with body condition and pregnancy status in Dall’s sheep across all the analyses performed. The intensity of the intestinal whipworm, *Trichuris schumakovitschi*, decreased with age. No other parasites were significantly associated with age, body condition, or pregnancy. Our study suggests that *M*. *marshalli* might negatively influence fitness of adult female Dall’s sheep.

## Introduction

It is widely accepted that nematode parasites can have a subtle but significant detrimental effects on host fitness[1–3]. In wild ungulates, for instance, experimental and observational work has shown that gastrointestinal helminths can negatively influence pregnancy rates[4,5], body condition[5–8], offspring survival[9] and mortality[9–11]. The study of these effects in wild ungulates from Arctic and Subarctic regions is important for a variety of reasons: i) human communities in these regions depend on wild ungulates as a source of food, as economic drivers through sport hunting and tourism, and as a focus for cultural activities[12], ii) Arctic and Subarctic regions are among the areas with least human disturbance globally, however, the current and predicted climate change is more prominent in high latitudes causing rapid and deep ecological changes and altering host-parasite dynamics[13–15], iii) wild animal and parasite communities are comparatively more simple than in temperate and tropical systems decreasing the number and complexity of potential confounding factors, and thus, serving as an ideal model for testing ecological mechanisms[4,16]

The Dall’s sheep (*Ovis dalli dalli*) is a wild ungulate endemic to the arctic and subarctic regions of North America, including Alaska (AK, United States), Yukon (YT, Canada), and Northwest Territories (NT, Canada)[17]. It breeds in late November and early December with births occurring the following April-May. During the short summer, ewes seek solitude and protection in rugged terrain for growth and development of lambs and to recover their body reserves[18,19]. Dall’s sheep are an important subsistence species for northern indigenous people as well as a valuable sport hunting prize, appreciated for their high-quality meat and large horn size[20,21].

The parasitological information on Dall’s sheep is limited to serological surveys of microparasites like *Toxoplasma gondii*[22], description of *Eimeria* spp.[23], detailed taxonomic and ecological studies on the protostrongylid nematodes *Parelaphostrongylus odocoilei* and *Protostrongylus stilesi*[24–26], and broad-based fecal and gastrointestinal parasite surveys[27–30]. The nematode *Marshallagia marshalli* has been documented as the gastrointestinal macroparasite with the highest infection intensities and prevalence throughout Dall’s sheep distribution range[28–30]. The pathophysiological changes caused by *M*. *marshalli* include a marked loss of functional parietal cells in the abomasum of the host resulting in an increase of abomasal pH, and an increase of both serum pepsinogen and serum gastrin concentrations[31,32]. The clinical presentation is similar to other gastrointestinal parasites including appetite depression, weight loss, constipation and diarrhea[31,33]. Based on field observations in Alaska, Neilsen and Neiland[29] suggested that *M*. *marshalli* might chronically weaken adult sheep and alter the digestion capacity of crude protein and dry matter in lambs during their first winter. Despite the importance of Dall’s sheep for the local economy and subsistence, plus the mounting evidence on the detrimental effect of parasites on wild ungulates in arctic and subarctic regions[4,5,34], little is known about the impacts of gastrointestinal helminths and particularly *M*. *marshalli* on Dall’s sheep.

The objectives of this study were to describe the diversity of gastrointestinal parasites in Dall’s sheep and to determine the relationship between parasite intensity and fitness indicators. To do this, we used an unprecedented historical collection of parasites and associated data in Dall’s sheep collected from the Mackenzie Mountains, Canada in 1971–1972. We had three specific objectives; to (i) describe the diversity and intensity of gastrointestinal helminths, and their relationship with age classes and sex, (ii) determine the association between *M*. *marshalli* intensity and body condition indicators, and finally, (iii) determine the association between infection intensity of *M*. *marshalli* and pregnancy status in adult females Dall’s sheep.

## Material and methods

### The simmons collection

From 1968 to 1972 an extensive scientific collection of Dall’s sheep occurred as part of a demographic study of sheep in the Mackenzie Mountains, Northwest Territories (NWT), Canada[35]. The two largest collections occurred in the winters of 1971 and 1972 and, in addition to the physiological and reproductive data, gastrointestinal parasites were collected by Dr. Anne Currier, a veterinarian for the Canadian Wildlife Service, Fort Smith, NWT. After preliminary evaluation in the early 1970s, no further work was done on the parasite collection and its whereabouts were unknown until the spring of 2000, when it was located as an orphaned collection at the Canadian Museum of Nature (CMN) in Ottawa (Ontario, Canada). The specimens and associated documentation were recovered and evaluated by Susan Kutz along with Eric Hoberg and Alasdair Veitch, who determined that it was an extremely valuable, unprecedented intact dataset and parasite specimen collection for Dall’s sheep. It is now known as “The Simmons Collection”.

**Fig 1:**
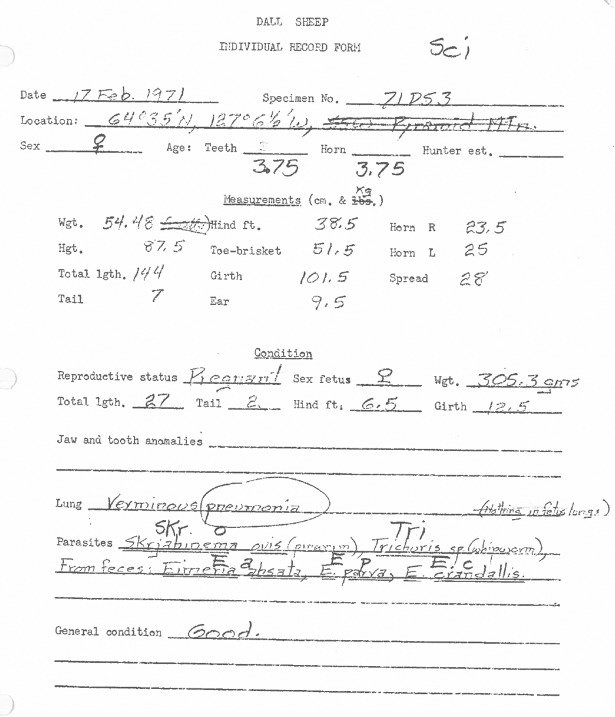
Original post mortem card. Card used by Dr. Norman Simmons to record the individual physiological and reproductive information of Dall’s sheep captured in the Mackenzie Mountains during February 1971–1972.

The collection consisted of four boxes containing approximately 100 vials of parasites each, as well as a small collection of specimens mounted on slides. All the documentation was stored in a single binder containing individual cards with the parasitological information from each sheep and an extensive scientific correspondence among the principal investigators involved in this research, including Dr. Norman Simmons (project leader, Fort Norman), Dr. Anne Currier (veterinarian, Fort Smith, post mortem examinations, isolation and quantification of helminths), Dr. Laurel P. E. Choquette (Ottawa, identification of helminths), Dr. Eric Broughton (veterinary pathologist, Ottawa, lung pathology), and Dr. G. G. Gibson (Ottawa, involved in the lungworm studies). In addition, Dr. Norman Simmons provided the original post mortem cards (Fig 1) and summary sheets with the information on physiological and reproductive parameters from each sheep collected in 1971 and 1972 (see below).

### Dall’s sheep captures and data collection

The Dall’s sheep collection occurred in February 1971 and 1972 from several independent subpopulations inhabiting the areas of Mountain and Moosehorn rivers in the north central Mackenzie Mountains (65 degrees N; 128 degrees W), Canada. The captures were performed with a high-powered rifle fired from a helicopter, including all sheep in the first group encountered except for those clearly identifiable as rams. The information on physiological and reproductive parameters on each sheep was recorded on individual post-mortem cards and summary sheets[35]. The post mortem cards included information on sex (i.e. male, female), age in years based on both counts of horn rings and tooth cementum annuli, reproductive status determined by the presence or absence of a foetus, body weight (Kg), several body measurements (e.g. total body length, chest circumference) and a body condition score based on the presence/absence of abdominal and back fat (Fig 1).

The gathering of specimens and data on gastrointestinal parasites was performed by Dr. Anne Currier. Adults worms from the abomasum, small intestine, large intestine and caeca of each sheep were isolated from each organ, provisionally identified, counted, and stored in 10% formalin. Between 2003–2005, S. Kutz, together with EP. Hoberg, retrieved these vials, rehydrated the specimens when needed, and then mounted them on microscope slides and cleared them in 5% glycerol for subsequent examination and identification at the species level according to [36–42].

### Data analyses

#### Sheep body condition

We used two different approaches to assess body condition of the sheep: i) a categorical body condition score (BCS) based on the presence or absence of subcutaneous fat reserves, and ii) a non-destructive residual index based on mass and morphometric measurements (scale mass index, SMI)[43].

The BCS was determined in the field and recorded in the post mortem cards. Sheep with no abdominal or back fat were classified as ‘Lean’; sheep with abdominal but not back fat were classified as ‘Fair’; sheep with abdominal and some back fat were classified as ‘Good’, and sheep with much or good back fat were classified as ‘Very Good’.

The SMI is a recent innovation that standardizes body mass at a fixed value of a linear body measurement based on the scaling relationship between mass and length[43,44]. This is a versatile index as it is independent from body size, sex, and physiological status, and unlike most residual indices, it can be used to compare across populations[45]. The SMI was calculated according to [43] as shown in the following formula:

$$SMI=M_{i}{\left[\frac{L_{0}}{L_{i}}\right]}^{b_{SMA}}$$ (1)

where *M*_*i*_ is the body mass and *L*_*i*_ is the body length of individual *i*, the *b*_*SMA*_ is the regression coefficient in the Standardized Major Axis regression of ln-transformed *M* on ln- transformed *L*, and *L*_*0*_ is an arbitrary value of *L*. Normally the arbitrary value used is the arithmetic mean of *L* for the sample under study. Finally, *SMI* is the predicted scale mass index for individual *i*. In the case of pregnant females, we corrected their weight by subtracting the weight of their fetus (mean fetuses weight: 0.236 kg, SE = 0.013) before calculating their SMI.

#### Parasite intensity, prevalence, and species dominance

We determined the intensity of infection (i.e. median number of worms) and prevalence (i.e. percentage of positive cases + 95% confidence intervals) for each species of gastrointestinal helminth by sex, pregnancy status and year of collection. In the case of infection intensity and prevalence by sheep age, we used two different approaches: i) to compare juveniles with adults we used two age categories; juveniles (<24 months of age) and adults (> 24 months of age), and ii) to determine the age pattern of infection for the six most abundant parasite species we used five age categories: lambs (<1 year), yearlings (1 year old), young adults (2 to <6 years), mature adults (6 to <11 years) and old adults (11 years and older). In all the analyses, the differences in infection intensity between categories were explored using a t-test with Welch Correction to account for differences in variance. In the cases where the assumption of normality was not met a permutation t-test with 10,000 permutations was used. The differences in parasite prevalence among categories were determined using chi-squared test with Monte-Carlo significance test procedures (2000 replicates) to account for low cell counts (<5) in the contingency tables. To test differences in parasite infection intensity by BCS we used a one-way ANOVA and log+1 transformed parasite intensity to account for normally distributed residuals. The Tukey HDS test was used for pairwise comparisons. In the univariate analyses described above, all the observations available for each species were used.

The species dominance among gastrointestinal parasites was determined by using two approaches: a Dominance Index (DI) and a Dominance Value (DV). The DI, originally proposed by [46], considers species prevalence and is calculated by multiplying the total number of species *i* by the number of hosts infected with species *i* and divided by the total number of hosts examined squared. In this index, a dominant species was defined as one in which the DI is greater than 1, and a co-dominant species the one which the DI value was between 0.01 and 0.99. The DV, originally proposed by [47], considers the total number of parasites in the entire host sample. DV is calculated simply by dividing the total number of species *i* in the entire host sample by the total number of gastrointestinal parasites including all the species in the host sample, and multiplying the result by one hundred. Only the sheep with complete information for all the parasite species were included in the calculation of both approaches in order to obtain comparable results among species.

#### Fitness indicators and gastrointestinal parasites

The relationship between fitness indicators in the host (i.e. BCS, SMI and pregnancy), and the infection intensity of gastrointestinal helminths was explored using two approaches: i) Generalized Linear Models (GLM), this approach provide a flexible framework to study the association of continuous (i.e. SMI) and discrete (i.e. BCS and pregnancy status) outcomes and a set of independent variables. However, the number of predictor variables in the candidate model is directly limited by, among other factors, sample size (e.g. >10 outcome events per predictor variable) [48]. The models with too few outcome events relative to the number of predictor variables might produce biased estimates of regression coefficients [49], and ii) Partial Least Squares Regression (PLS-R) provides a flexible framework to study the association of continuous variables (i.e. SMI) and a set of independent variables. This approach is particularly useful when the number of sample units is low with respect to the number of measured independent variables [50]. Specifically, the PLS-R was used to investigate the simultaneous effect of helminths co-infection in host fitness.

The GLMs were fitted as follows: in the case of BCS we used ordinal logistic regression to account for the order on the categories in the outcome variable. Proportional odds ratios were obtained by the exponentiation of the coefficients from the independent variables in the final model. In order to meet the sample size requirements per predictor variable, the parameters included were reduced to: infection intensity of only the helminth species negatively associated with fitness indicators in the univariate analyses (i.e. *M*. *marshalli* and *T*. *schumakovitschi*), infection intensity of all the intestinal parasite species combined, age in years and pregnancy status. The SMI was analyzed using a GLM with a Gaussian error structure and a log link function and the parameters included in the models were the same as in the analyses of BCS. Finally, a GLM with binomial errors structure and a log link function was used to analyze pregnancy status in adult female sheep using the same parameters included in the analyses of BCS plus body condition (i.e. SMI). In order to facilitate the interpretation of coefficients and proportional odds ratios in all the models fitted, parasite intensity was divided by one hundred before being included in the models. By doing this we re-scaled the independent variable to a more meaningful unit from one parasite to one hundred parasites. In order to include pregnancy status as an independent variable, only adult females were included in the analyses. In all the cases when GLMs were used the model comparison was done using Akaike Information Criterion (AIC)[51].

In the case of the PLS-R, the independent variables included in the model were the intensity of each gastrointestinal parasite species (nine species), the species richness (i.e. number of parasite species per host), age in years, and pregnancy status, resulting in a total of 12 independent variables. The single dependent variable was SMI. In order to include pregnancy status as an independent variable, only adult females were included in the analysis. Both GLMs and PLS-R were performed with the R statistical software (R Development Core Team) and only sheep with complete information in all the independent variables were included. A significance level of p < 0.05 was used in all the analyses.

## Results

### General results

A total of 112 Dall’s sheep were collected from the Mackenzie Mountains during 1971 and 1972 [35]. However, The Simmons Collection at the CMN contained parasitological information only from 104 animals (23 from 1971 and 81 from 1972); 88 adult females (20 from 1971 and 68 from 1972), 6 yearling females (1 from 1971 and 5 from 1972), 8 yearling males (2 from 1971 and 8 from 1972) and two male lambs captured in 1972. It was not possible to determine the whereabouts of the information on the 8 remaining sheep.

Prevalence of gastrointestinal helminths was 100%. Eight different nematode species were identified; *Marshallagia marshalli* and *Ostertagia gruehneri* collected from the abomasum; *Nematodirus andersoni*, *Nematodirus davtiani*, *Nematodirus oiratianus interruptus*, *Nematodirus spathiger*, and *Trichostrongylus* sp. collected from the small intestine; and *Trichuris schumakovitschi* and *Skrjabinema ovis* collected from the large intestine (cecum and colon). In addition, the cestode *Moniezia* sp. was identified in the small intestine. There were no differences in parasite prevalence and intensity between 1971 and 1972 for any of the parasite species analyzed, total count of intestinal parasites or total gastrointestinal parasites (S1 Table). For this reason, the data in the rest of manuscript were analyzed and presented grouping both years. Information on abomasal helminths species was available only for 79 sheep. Total count of *Nematodirus* spp. was available for 102 sheep but *Nematodirus* species identification were only available for 54 sheep. *Marshallagia marshalli* was the most common parasite species among all sheep with the highest prevalence (100%, n = 79, 95% Confidence Interval [95% CI] = 94.1–100) and highest intensity of infection (median = 224, range 22–2457). *Marshallagia marshalli* is a dimorphic species with two distinct male forms [37]. The minor morphotype, designated as *Marshallagia occidentalis*, was not observed in the Simmons collection. Counts of *M*. *marshalli* reflected in the current paper would thus refer solely to the major morphotype. *Nematodirus* spp. were detected in 95% (n = 102, 95% CI = 88.2–98.1) of the sheep although with significantly lower intensity of infection (median = 98, range 1–1427) than *M*. *marshalli* (Permutation test, Z = 3.074, p-value<0.01). Among all *Nematodirus* spp., *Nematodirus spathiger* had the higher prevalence (94.4%, n = 54, 95%CI = 83.7–98.6) and infection intensity (median = 78, range 2–485). For more details see Table 1. *Marshallagia marshalli* was the species with the highest dominance index and dominance value, followed in both cases, by *N*. *spathiger* and *N*. *oiratianus interruptus*. These dominance indicators were several times higher in *M*. *marshalli* compared to any other species in both juvenile and adult sheep (Table 2).

**Table 1:** Prevalence and median infection intensity of gastrointestinal helminths in Dall´s sheep categorized as Juveniles (sheep younger than 24 months, including two lambs younger than 12 months) and adults (sheep 24 months and older).

	Juveniles				Adults			
	n	No. of infected	Prevalence % (95% CI)	Median (Range)	n	No. of infected	Prevalence % (95% CI)	Median (Range)
**Abomasal parasites**								
***Ostertagia gruehneri***	13	0	0 (0–28.3)	NA	66	1	1.5% (0–9.3)	360 (1)
***Marshallagia marshalli***	13	11	100 (67.8–100)	252 (22–853)	66	66	100 (93.1–100)	223.5 (42–2457)
**Small intestine parasites**								
***Moniezia* sp.**	16	2	12.5 (2.2–39.6)	1.5 (1–2)	86	13	15.1 (8.6–24.8)	1 (1–3)
***Trichostrongylus* spp.**	16	0	0 (0–24.1)	NA	86	2	2.3 (0.4–8.9)	2 (1–3)
***Nematodirus andersoni***	12	7	58.3 (28.6–83.5)	13 (3–66)	42	21	50 (35.5–64.5)	27 (2–189)
***Nematodirus davtiani***	12	6	50 (25.3–74.6)	29.5 (5–176)	42	13	30.9 (18.1–47.2)	19 (3–490)
***Nematodirus oiratianus interrruptus***	12	9	75 (42.8–93.3)	119 (20–431)	42	25	59.5 (43.3–73.9)	67 (4–469)
***Nematodirus spathiger***	12	11	91.6 (59.8–99.6)	109 (6–202)	42	40	95.2 (82.6–99.2)	73 (2–485)
**Large intestine parasites**								
***Trichuris schumakovitschi***	16	16	100 (75.9–100)	29.5 (5–210)	86	75	87.2 (77.9–93.1)	14 (1–131) *
***Skrjabinema ovis***	16	6	37.5 (16.3–64.1)	18.5 (1–68)	86	55	63.9 (52.8–73.8)	5 (1–45) *
**Parasites grouped**								
**Total *Nematodirus* spp.**	16	16	100 (75.9–100)	232.5 (4–630)	86	81	94.1 (86.3–97.8)	87 (1–1427)
**Total Intestinal parasites**	16	16	100 (75.9–100)	312 (45–643)	86	86	100 (94.7–100)	122.5 (2–1481)
**Total Gastrointestinal**	13	13	100 (71.7–100)	516 (173–1234)	66	66	100 (93.1–100)	414.5 (65–3715)

Significant differences in intensity between age classes are indicated by * (*p*<0.05, Randomization test).

No differences in prevalence between age classes were found in any of the species or groups of parasites (*p*<0.05, chi-squared test).

**Table 2:** Species dominance of gastrointestinal helminths in Dall´s sheep categorized as Juveniles (sheep younger than 24 months, including two lambs younger than 12 months) and adults (24 months and older).

	Juveniles (n = 12)		Adults (n = 39)	
	Dominance Index	Dominance Value	Dominance Index	Dominance Value
**Abomasal parasites**				
***Ostertagia gruehneri***	0	0	0.0002	0.01
***Marshallagia marshalli***	280.1*	47.9	478.5*	61.9
**Small intestine parasites**				
***Moniezia* sp.**	0.03	0.04	0.023	0.02
***Trichostrongylus* spp.**	0	0	0.005	0.01
***Nematodirus andersoni***	7.8*	2.3	14.7*	3.7
***Nematodirus davtiani***	13.4*	4.6	11.9*	4.6
***Nematodirus oiratianus interruptus***	89.4*	20.4	40.9*	9.4
***Nematodirus spathiger***	96.9*	16.6	124.7*	17.0
**Large intestine parasites**				
***Trichuris schumakovitschi***	47.7*	8.2	18.2*	2.6
***Skrjabinema ovis***	0.15*	0.09	2.24*	0.6

Species classified as Dominant (Dominance Index >1) are indicated by *.

### Distribution of gastrointestinal parasites by age and sex

**Fig 2:**
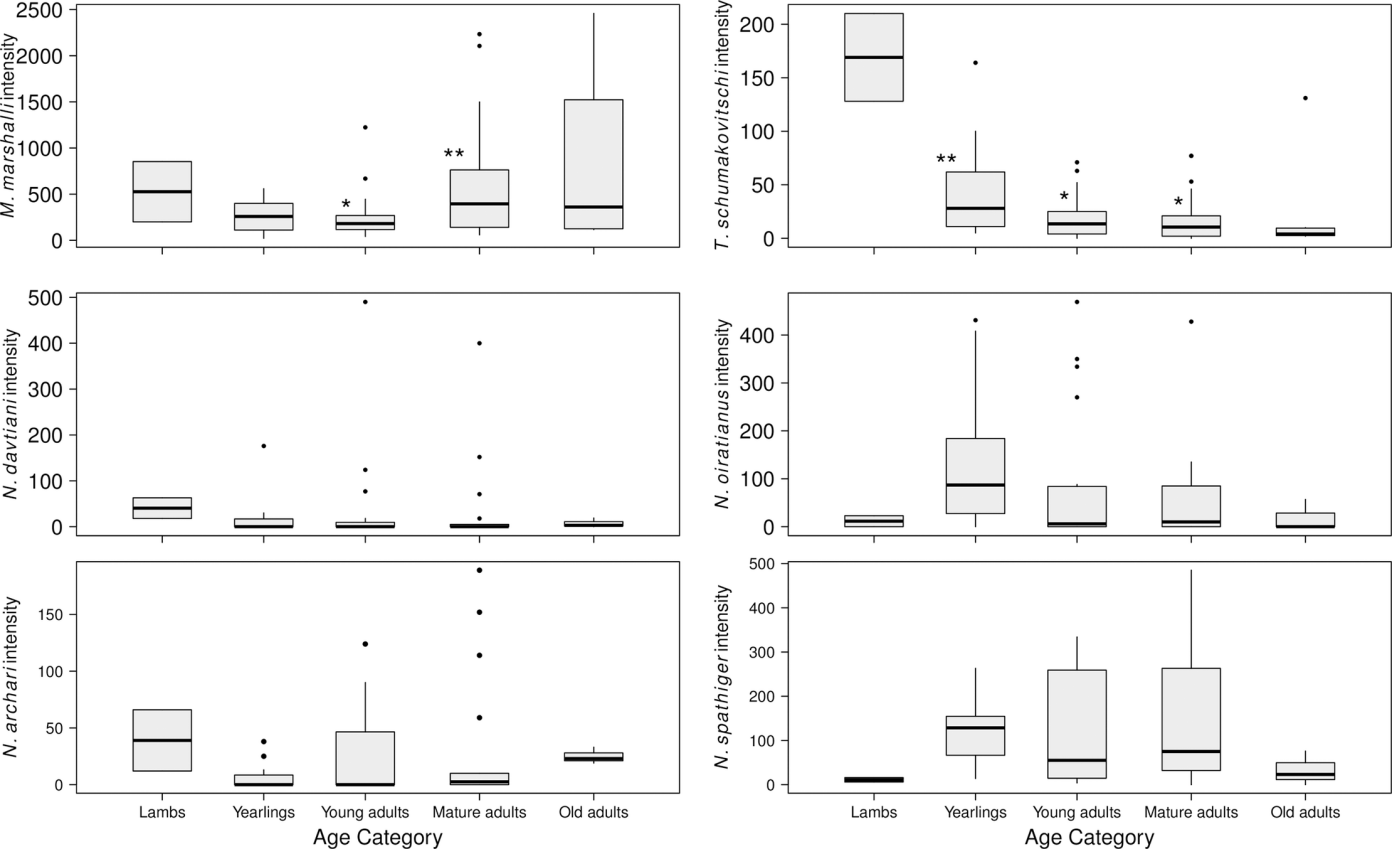
Infection intensity of gastrointestinal helminth species in adult Dall’s sheep categorized by age class. Lambs and old adults were excluded from pairwise comparison because of small sample size (n<5). Significant difference among categories (p<0.05) are represented with “*”.

We analyzed the age distribution pattern of infection intensity for the six most abundant parasite species (i.e. five age classes of the host): *M*. *marshalli*, *N*. *andersoni*, *N*. *davtiani*, *N*. *oiratianus interruptus*, *N*. *spathiger* and *T*. *schumakovitschi* (Fig 2). *Trichuris schumakovitschi* had a significantly higher infection intensity in immature sheep (Permutation test, Z = -2.265, p-value = 0.023), with yearlings having significantly higher infection intensity than young adults (Pairwise permutation test, W = -3.081, p-value = 0.012) and mature adults (Pairwise permutation test, W = -2.851, p-value = 0.013). The opposite trend was observed for *M*. *marshalli*, with higher infection intensity in mature adults than in young adults (Pairwise permutation test, W = 2.681, p-value = 0.0219). The infection intensity among the remaining parasite species did not differ among age classes. Comparison of infection intensity between sexes was done only in yearlings as no older males were captured. The infection intensity of *T*. *schumakovitschi* in male sheep (n = 8, median = 65, range 11–210) was significantly higher than in females (n = 8, median = 16.5, range = 5–62) (Permutation test, Z = -1.84, p-value = 0.021). There was no difference of infection intensity between sexes for other parasite species.

### Infection intensity of parasites and body condition

**Fig 3:**
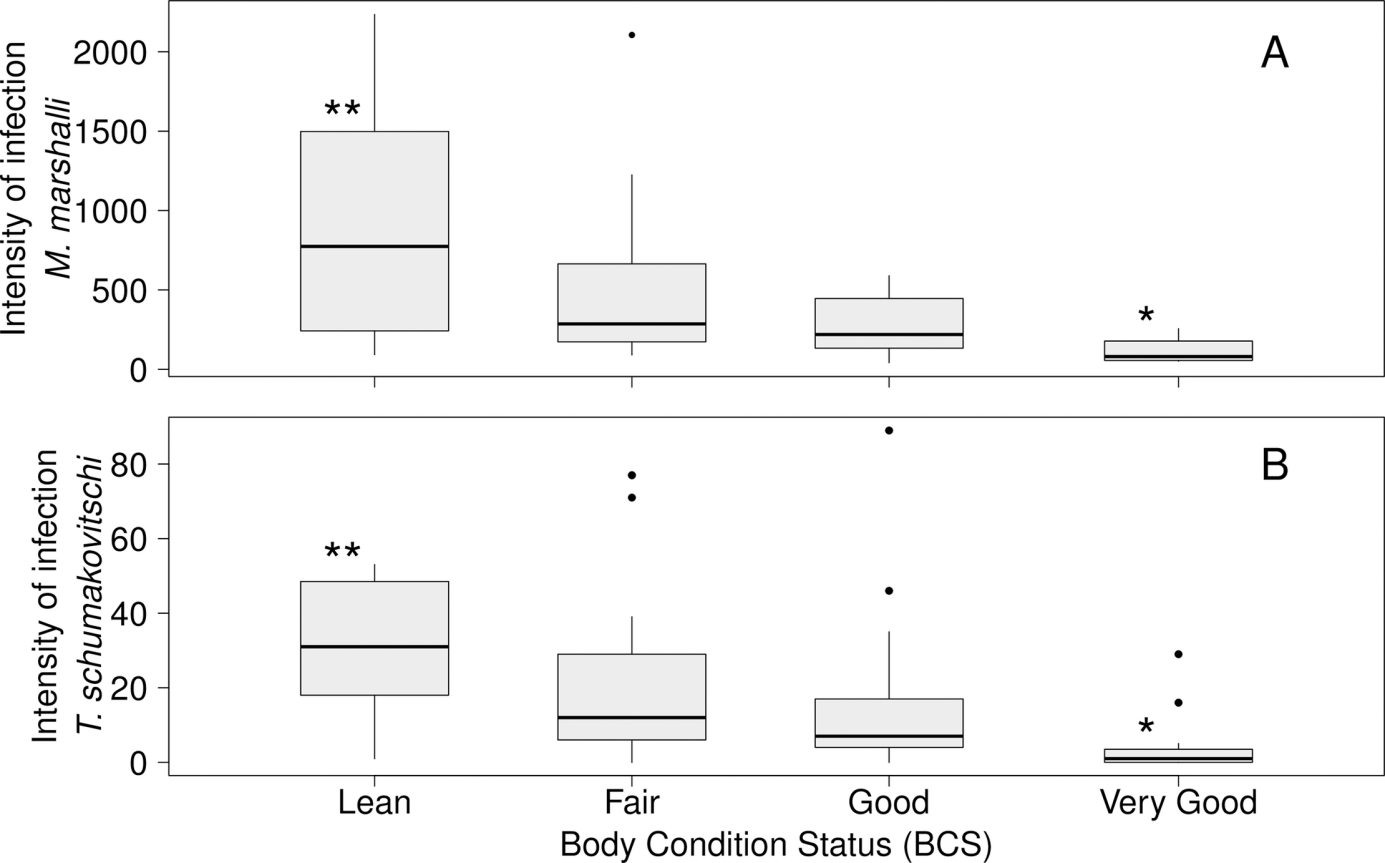
Infection intensity of gastrointestinal helminths in adult Dall’s sheep categorized by body condition (BCS). A) Total infection intensity of *M*. *marshalli* (Pairwise comparisons, n = 42, Lean-Very Good, W = -2.674, p = 0.0249). B) Infection intensity of *T*. *schumakovitschi* (Pairwise comparisons, n = 61, Lean-Very Good, W = -2.816, p = 0.0291). Significant differences among categories (p<0.05) are represented with “*”.

**Fig 4:**
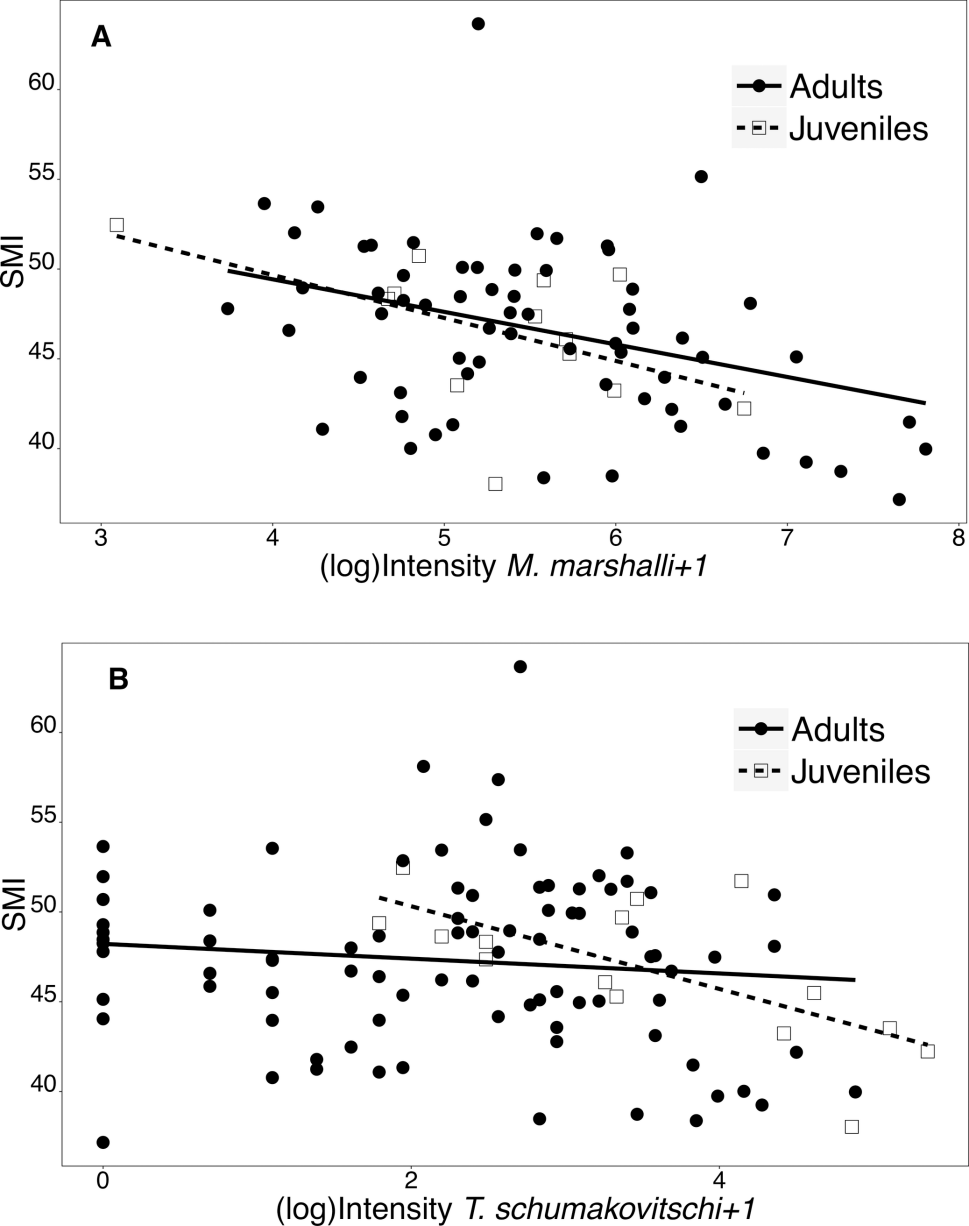
Relationship between SMI and infection intensity of parasites in Dall’s sheep. The lines and dashed lines represent the predicted SMI for adults and yearling Dall’s sheep, respectively, estimated by a linear regression. A) *Marshallagia marshalli* intensity (Linear regression, Adults (n = 66): coefficient = -1.813, p = 0.004, R^2^ = 0.124; Yearlings (n = 13): coefficient = -2.392, p = 0.062, R^2^ = 0.28) and B) *Trichuris schumakovitschi* intensity (Linear regression, Adults (n = 86): coefficient = -0.411, p = 0.311, R^2^ = 0.012; Yearlings (n = 16): coefficient = -2.302, p = 0.004, R^2^ = 0.434).

The body condition of adult ewes was negatively associated with the infection intensity of gastrointestinal parasites. Information in BCS was available only for 63 adults and 12 juveniles. The total count of gastrointestinal helminths (log_10_-transformed) in adult females with both Lean and Fair BCS was significantly higher than in sheep in Very Good BCS (ANOVA, F_3,38_ = 6.965, p< 0.001). For individual parasites species, only the intensity of *M*. *marshalli* and *T*. *schumakovitschi* was negatively associated with BCS (*M*. *marshalli*, n = 42: Permutation test, Z = -2.966, p-value = 0.0013, Fig 3A; *T*. *schumakovitschi*, n = 61: Permutation test, Z = -2.756, p = 0.005, Fig 3B). The infection intensity of *M*. *marshalli* (median = 223.5, range = 42–2457) was significantly higher than *T*. *schumakovitschi* (median = 14, range = 1–131) (Permutation test, p<0.001). The BCS in juvenile sheep was not associated with either total count of gastrointestinal helminths, *M*. *marshalli* or any parasite species. However, the sample size was low and there was an unbalanced distribution among BCS categories. With respect to SMI, adult Dall’s sheep with lower SMI had significantly higher infection intensities of *Marshallagia marshalli* (Fig 4). Juvenile sheep also had the same pattern of higher infection intensity of *M*. *marshalli* with lower SMI, however, the association was barely not significant. *Trichuris schumakovitschi* also had a negative association with SMI in juvenile sheep (Fig 4). No other single species, nor total intestinal parasites as a whole, were negatively associated with SMI.

A similar negative association between body condition indicators and the intensity of gastrointestinal parasites was observed when data were analyzed using a multivariate approach. In the case of BCS, the best model included *M*. *marshalli* infection intensity/100 (log odds ratio = 0.83, 95% CI = 0.71 0.95, Table 3) and the infection intensity of intestinal parasites/100 (log odds ratio = 0.78, 95% CI = 0.61 0.96, Table 3). *Marshallagia marshalli* was present in the first six models with the lowest AIC and always had a significant negative effect on BCS regardless of the other variables included in the model (S2 Table). Similarly, the best model describing SMI in Dall’s sheep included the effects of *M*. *marshalli* infection intensity and *T*. *schumakovitschi* (Table 3). The first nine models with the lowest AIC contained the effect of *M*. *marshalli* infection intensity (S2 Table). The effect of pregnancy status was also included in the best model although its effect on SMI was non-significant (Table 3).

**Table 3:** Final Generalized Linear Models for BCS, SMI and pregnancy status of adult female Dall’s sheep from Mackenzie Mountains.

Model	Approach	n	Fixed effects	Estimate	SE	*P-value*	95% CI
BCS Dall’s sheep	Ordinal regression	42	*M*. *marshalli* intensity/100	-0.1807	0.072	0.013	-0.335–0.047
			Intestinal parasites intensity/100	-0.2190	0.169	0.049	-0.484–0.010
SMI Dall’s sheep	Gaussian	66	*M*. *marshalli* intensity/100	-0.0074	0.0028	0.014	-0.013–0.0018
			*T*. *schumakovitschi* intensity/100	-0.0564	0.0612	0.360	-0.176 0.0608
Pregnancy status	Binomial	66	*M*. *marshalli* intensity/100	-0.1391	0.0594	0.0191	-0.273–0.032

In the PLS-R analysis, where 39 adult females had complete information for all parasite species, the first component explained a 23.22% (R^2^) of the original variance in the SMI and the second component explained 8% of the remaining unexplained variance (Table 4). The remaining 12 components included in the analysis had negligible importance in explaining SMI. In component one, *M*. *marshalli*, *N*. *archari* and *T*. *schumakovitschi*, in decreasing order, had the greatest negative effect on SMI and explained 76.98% of the information. Interestingly, either *N*. *andersoni* or *T*. *schumakovitschi* were significantly associated with SMI in univariate analyzes in the same dataset (Linear Regression; *N*. *andersoni* (n = 39), coefficient = -0.030, p = 0.079, R^2^ = 0.081; *T*. *schumakovitschi* (n = 39), coefficient = -0.055, p = 0.133, R^2^ = 0.059). In component two, *M*. *marshalli* and *N*. *spathiger* explained 84.91% of the component but only *M*. *marshalli* was negatively associated with SMI.

**Table 4:** Results of the Partial Least Squares Regression (PLS-R) explaining the effects of several gastrointestinal parasite species on body condition in adult female Dall’s sheep (n = 39).

	Component 1		Component 2	
	Weight	% Variance explained	Weight	% Variance explained
Parasite species				
*Nematodirus andersoni*	**-0.399**	**15.94**	-0.065	0.43
*Nematodirus davtiani*	-0.103	1.06	0.267	7.12
*Nematodirus oiratianus interruptus*	-0.154	2.38	0.251	6.30
*Nematodirus spathiger*	0.034	0.11	**0.553**	**30.63**
*Trichuris schumakovitschi*	**-0.343**	**11.77**	-0.083	0.68
*Skrjabinema ovis*	-0.211	4.44	-0.255	6.54
*Moniezia* sp.	-0.171	2.91	0.211	4.48
*Trichostrongylus* spp.	-0.145	2.12	0.290	8.41
*Marshallagia marshalli*	**-0.701**	**49.25**	**-0.737**	**54.28**
Age	-0.161	2.58	-0.170	2.89
Parasite diversity	-0.253	6.44	0.173	2.99
Pregnancy	0.095	0.91	-0.225	5.07
**R**^**2**^	0.232		0.080	

Weights of each variable in the first and second PLS-R components and R^2^ representing the variance in the response variable accounted for in each component in the PLS-R.

### Infection intensity of parasites and pregnancy status

**Fig 5:**
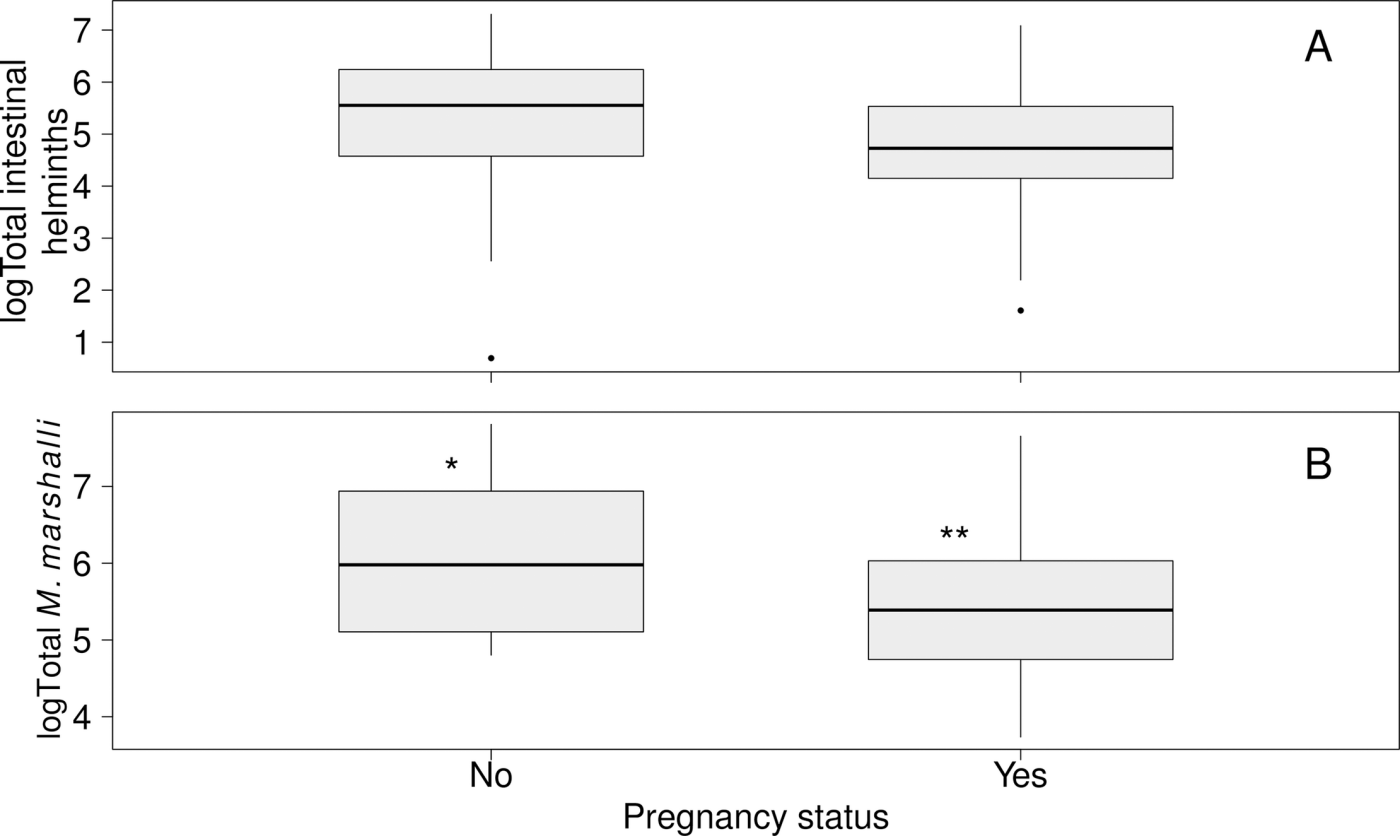
Infection intensity of gastrointestinal helminths in adult female Dall’s sheep categorized by pregnancy status. A) Total infection intensity of intestinal helminths (t-test, t = 1.598, df = 25.96, p-value = 0.122). B) Infection intensity of *Marshallagia marshalli* (: t-test, t = 2.444, df = 24.753, p-value = 0.0220). Significant difference among categories (p<0.05) are represented with different numbers of “*”.

Pregnancy was negatively associated with the infection intensity of gastrointestinal parasites in adult female Dall’s sheep (Fig 5). This negative relationship seemed to be largely determined by *M*. *marshalli*; pregnant sheep had significantly lower log_10_-*M*. *marshalli* infection intensity than non-pregnant sheep (Fig 5, Table 5). There were no statistically significant associations of pregnancy with other individual species or intestinal parasites as a whole (Fig 5). The age (in years) of pregnant (Median = 5.8, range = 2.8–11.8) and non-pregnant (Median = 6.8, range = 2.8–12.8) ewes was not significantly different (Permutation test, Z = 0.556, p-value = 0.6181). The model with the better-fit explaining pregnancy in Dall’s sheep included only the effect of *M*. *marshalli* infection intensity (Table 4). There was a significant negative effect of *M*. *marshalli* infection intensity on Dall’s sheep pregnancy rate (estimated log odds ratio per 100 adult *M*. *marshalli* = 0.87, 95% CI = 0.76 0.97).

**Table 5:** Infection intensity and prevalence of gastrointestinal helminths in Dall´s sheep categorized by pregnancy status.

	Non Pregnant				Pregnant			
	n	No. of infected	Prevalence % (95% CI)	Median (Range)	n	No. of infected	Prevalence % (95% CI)	Median (Range)
**Abomasal parasites**								
***Ostertagia gruehneri***								
***Marshallagia marshalli***	17	17	100 (77.1–100)	395 (122–2457)	49	49	100 (90.6–100)	219 (42–2106)*
**Small intestine parasites**								
***Moniezia* sp.**	19	4	26.6 (6.9–46.1)	1 (1–1)	67	9	13.4 (6.6–24.5)	1 (1–3)
***Trichostrongylus* spp.**	19	1	5.2 (0.2–28.1)	1 (1)	67	1	1.5 (0.08–9.1)	3 (3)
***Nematodirus andersoni***	9	5	55.6 (22.7–84.7)	66 (5–189)	33	16	48.5 (31.2–66.1)	23 (2–152)
***Nematodirus davtiani***	9	3	33.3 (9.1–69.1)	18 (11–400)	33	10	30.3 (16.2–48.9)	45 (3–490)
***Nematodirus oiratianus interruptus***	9	8	88.9 (50.7–99.4)	48.5 (4–428)	33	17	51.5 (33.9–68.8)	67 (4–469)
***Nematodirus spathiger***	9	9	100 (62.9–100)	144 (6–485)	33	31	93.9 (78.4–98.9)	55 (2–473)
**Large intestine parasites**								
***Trichuris schumakovitschi***	19	18	94.7 (71.9–99.7)	15 (1–131)	67	57	85.1 (73.8–92.2)	14 (1–89)
***Skrjabinema ovis***	19	11	57.8 (33.9–78.9)	2 (1–24)	67	44	66.7 (52.9–76.6)	5.5 (1–45)
**Parasites grouped**								
**Total *Nematodirus* spp.**	19	19	100 (79.1–100)	220 (1–1427)	67	62	92.5 (82.7–97.2)	58.5 (1–1158)
**Total Intestinal parasites**	19	19	100 (79.1–100)	258 (2–1481)	69	67	97.1 (88.9–99.5)	83 (5–1191)
**Total Gastrointestinal**	17	17	100 (79.1–100)	477 (165–3715)	49	49	100 (90.6–100)	364 (65–2200)

Significant differences in intensity between pregnancy status are indicated by * (*p*<0.05, Randomization test).

## Discussion

Analyzing the Simmons Collection allowed us to describe the diversity, intensity and prevalence of gastrointestinal helminths in Dall’s sheep from the Mackenzie Mountains in 1971–1972. The parasite fauna identified in this study was similar to that reported and summarized elsewhere in the Northwest Territories and Alaska[28–30,52]. By linking this parasitological data with reproductive and physiological parameters of the host we found strong evidence of a negative association between infection intensity of parasites and both host body condition and pregnancy status. These results were largely determined by the infection intensity of the nematode *M*. *marshalli*, suggesting that this species can have detrimental effects on Dall’s sheep fitness. We first discuss the species diversity, then impacts, and conclude with discussing the value of the Simmons collection and of data and sample archiving.

### Species diversity and distribution

The diversity of parasite species identified in our study is similar to what has been previously described in Dall’s sheep and other wild ungulates in North America[28,53]. Consistent with other trichostrongylines, *M*. *marshalli* is a dimorphic ostertagiine nematode with a direct life cycle that occurs in the abomasum of several wild ungulates in North America such as muskoxen (*Ovibos moschatus*), bighorn sheep (*Ovis canadensis*), thinhorn sheep (*Ovis dalli*), and pronghorn (*Antilocapra americana*), among others[37]. This species has remarkable ecological tolerance and resilience to persist in extreme environmental conditions. For instance, the eggs remain viable after extended freezing[28] and the infective stage (L3) is also tolerant to freezing temperatures, allowing transmission in the Arctic winter[54,55]. *Nematodirus* spp. are primarily nematodes infecting the small intestine, are commonly found in Arctic and Subarctic ungulates, and are well adapted to life at high latitudes[28,56]. The four *Nematodirus* species reported in our study are the same as previously observed in Dall’s sheep from various locations in Alaska[29,39]. It appears that *N*. *andersoni* is the correct name for nematodirines previously considered to represent another species, *N*. *archari*, according to Durette-Desset and Samuel [40]; until shown otherwise, *N*. *archari* is now considered to be limited in distribution to wild sheep from eastern Eurasia where it was originally described. The most prevalent and numerically dominant *Nematodirus* species seems to vary among Dall’s sheep populations which may be due to the interaction between differences in the natural history among parasite species (e.g. development and hatching strategies, specific seasonal development), the sampling strategies in each sheep population (e.g. season of sampling, parts of the intestine examined), and perhaps geographic locality reflecting complex histories of population fragmentation and isolation[29,57]. Species of *Trichuris* are commonly found in the large intestine of Dall’s sheep[29] and bighorn sheep[58], but apparently are rare in other sympatric wild ungulates such as caribou and muskoxen[28]. *Trichuris schumakovitschi* is the only species of *Trichuris* identified to the species level in Dall’s sheep. The pinworm *Skrjabinema ovis* is also common in the large intestine and caecum of Dall’s sheep, although at low to moderate prevalence and intensity[30]. This pinworm is also found in bighorn sheep and mountain goats, but rarely observed in caribou, moose and muskoxen[28,29,59–61]. Tapeworms of the genus *Moniezia* are considered rare in Dall’s sheep and *M*. *benedeni* is the only species previously reported[30]. Recent discovery of a complex of cryptic species attributed to *Moniezia* in the Arctic suggests that this species identification requires confirmation [62]. *Ostertagia gruehneri* was found in a single individual. This nematode is typically found in high numbers in the abomasum of the sympatric mountain woodland caribou, however, it is highly seasonal, being most abundant in the summer[63]. The Simmons Collection represents only winter parasite diversity and it is probable, based on previous fecal analyses, that *O*. *gruehneri* and other related strongyles (e.g. *Teladorsagia boreoarcticus*), may be more common in Dall’s sheep during the summer, particularly in populations with high sympatry with caribou and muskoxen[28].

### Impact of parasites on Dall’s sheep fitness

Our data demonstrated a negative association between the infection intensity of *M*. *marshalli* and both body condition and pregnancy status of Dall’s sheep. The association between *M*. *marshalli* and host fitness has been examined for caribou and reindeer with contrasting results. In a cross-sectional study, Steele [64] determined that body condition indicators (i.e. protein mass index, kidney fat index, carcass weight) of female barrenground caribou from Kangerlussuaq-Sisimiut, west Greenland, were negatively associated with *M*. *marshalli* intensity, however, contrary to our study, there was no association with pregnancy rate. Dall’s sheep, possibly relate on larger body mass and the related higher amount of ingesta to cope with nutritional requirements during pregnancy. On the other hand, in an experiment using a delayed release anthelmintic bolus to control parasite intensity, Carlsson et al. [65] found no association between *M*. *marshalli* and body condition indicators in live wild Svalbard reindeer. The differences in the seasonal dynamics of *M*. *marshalli* among hosts species and populations, and differences in the natural history of the hosts themselves, might account for the variations in these results. For instance, egg production and infection intensity by *M*. *marshalli* in Dall’s sheep occurs year-round, with a slight decrease in the summer (Kutz et al. unpublished data,[29], suggesting a sustained pressure on the host throughout the entire year. In addition, this sustained pressure might increase with age as the intensity of *M*. *marshalli* significantly increased with age in Dall’s sheep. Conversely, *M*. *marshalli* in Svaldbard reindeer presents a strong seasonal pattern peaking in late spring and decreasing almost to zero during summer months suggesting a shorter seasonal impact[66]. The differences in parasite diversity among host populations might be also a source for divergent results. In Svalbard reindeer the abomasal fauna is largely dominated year-round by *O*. *gruehneri*, with a comparatively moderate increase in *M*. *marshalli* intensity during winter months but practically absent during summer[5]. Conversely, in the caribou population from Kangerlussuaq-Sisimiut in west Greenland, *O*. *gruehneri* is absent and, as occurs in Dall’s sheep, *M*. *marshalli* is the more prevalent species and with the higher intensity[64].

*Marshallagia marshalli* seems to be a species capable of producing a strong cumulative detrimental effect in Dall’s sheep. *Marshallagia marshalli* is the gastrointestinal helminth with the highest prevalence and infection intensity throughout Dall’s sheep distributional range[28–30]. It is an important cause of parasitic gastroenteritis in wild and domestic sheep[31,67] and can cause marked alterations in the abomasal physiology of the infected host[31,33]. In Dall’ sheep, this detrimental effect may occur through a variety of mechanisms that have been extensively studied for related nematodes in domestic ungulates. These include appetite depression, detrimental changes on host gastrointestinal function, alteration of protein metabolism and/or inducing a nutritionally demanding immune response[68–70]. The magnitude of these effects might be also influenced by the strong seasonality in the Mackenzie Mountains. As winter progresses, the forage availability can be limited only to wind-blown areas as snow cover becomes packed and crusted and sheep cannot dig through it, exacerbating the deficient nutritional uptake of highly parasitized Dall’s sheep[71]. A similar interaction between parasite effect and limited forage periods occurred in the soay sheep/*Teladorsagia circumcincta* system on St. Kilda, Scotland. The intensity of *T*. *circumcincta*, another abomasal ostertagiine, was negatively associated with daily survival in sheep experiencing food shortage during severe winters. However, experimentally infected sheep with *ad libitum* diet had only minor pathology and 100% survival, supporting that the impact of *T*. *circumcincta* can be exacerbated by reduced food availability[11]. According to this hypothesis, the effects of *M*. *marshalli* in Dall’s sheep fitness are perhaps more easily detected during periods of nutritional stress, e.g., winter months, which is when our study was performed.

*Trichuris schumakovitschi* was also negatively associated with body condition, however, this association was not consistent across all the analyses. In domestic ungulates, the intensity and prevalence of *Trichuris* spp. tends to be higher in yearling and young individuals and clinical signs include chronic gastroenteritis and, in heavy infections, diarrhea, anorexia and weight loss[72]. The intensity and effects of *Trichuris* spp. are not known in free-ranging arctic ungulates but they also appear to be more important in young age classes. For instance, variable intensity of *T*. *ovis* in captive muskoxen have been regularly associated with diarrhea in calves[73]. In our study, the infection intensity of *T*. *schumakovitschi* in Dall’s sheep significantly decreased with age, suggesting a similar age distribution than in domestic animals and a higher impact in young sheep.

The negative effect of multiple gastrointestinal species (co-infection) on fitness indicators and the view of hosts as complex “ecosystems of parasites” need also be considered in Dall’s sheep. For instance, although *M*. *marshalli*, under a co-infection approach (PLS-R), was still the most important species associated with fitness indicators in adult ewes, the negative association of *N*. *andersoni* with body condition was unexpected and not observed in any of the previous univariate analyzes. This outcome could have been the consequence of complex interactions between top down regulations (e.g. immune response of the host) and bottom up regulations (e.g. competition for resources among parasite species) only visible under a co-infection approach. The study of these interactions and the price of neglecting parasite group/species when addressing the cost of parasites to host fitness is gaining increasing attention in the disease ecology community [74,75].

### The simmons collection

The research presented herein would not have been possible without the foresight and attention to detail of Dr. Norman Simmons and colleagues over 40 years ago. The Simmons Collection is an unprecedented source of parasitological and ecological information on Dall’s sheep that would be nearly impossible to replicate today. For a variety of biological, ethical and political reasons, the collection of a similar number of Dall’s sheep for scientific purpose would be unlikely to be approved by the public and wildlife management agencies[76]. The Simmons Collection not only represents a parasitological dataset with an intrinsic value to assess ecological questions, it also, and maybe even more importantly, highlights the relevance of proper archiving samples in accredited repositories for the study of wildlife diseases[77,78]. Archival collections are the window to study past geographic and genetic distribution of disease agents and also are a direct opportunity to maximize the impact of federal and private funds over time[14,79–81].

On the other hand, there are also some limitations with respect of The Simmons Collection. First, there is no information on diversity and intensity of parasite larval stages in the sheep. Evidence from domestic[82] and wild[7,28] ungulates suggest that larval stages of ostertagiine nematodes in the abomasal mucosa can cause significant pathology and clinical effects on the host, particularly as a consequence of the mechanical damage when they emerge from the mucosa (e.g. Type II Ostertagiasis). Second, since the parasite specimens were preserved in formalin they are not suitable for molecular studies due to the highly fragmented nature of the DNA, even with the advent of nexgen methodologies. Recent studies suggest it may be possible to extract and sequence DNA from formalin-fixed specimens for use in phylogenetic analyses but the results still are highly variable and largely unreliable depending on specimen age and preservation conditions[83]. Finally, the sampling period in both years was restricted to February. Since the prevalence, intensity and composition of parasite communities is highly influenced by season[28], the information possible to obtain from The Simmons Collection on the life history of the parasites and parasite populations dynamics is limited to interpretation within the context of the season collected.

In conclusion, there is mounting evidence showing that gastrointestinal parasites can have strong influence at individual and population levels for wild ungulates[4–7,10,11,64,84]. In the case of Dall’s sheep, our study shows that *M*. *marshalli*, the most prevalent and numerically dominant species, might negatively influence fitness of their hosts by decreasing both body condition and pregnancy rates. Further research is needed to confirm and quantify the magnitude of this effect and to understand the main physiological, behavioural and ecological mechanisms involved. Further the Simmon’s Collection and the large collections of helminths in Dall’s sheep by Nielsen and Neiland (1974) from Alaska in the 1970’s are critical in establishing baselines to identify and document the outcomes of accelerating climate change and ecological perturbation in high latitude systems.

## Supporting information

S1 Table Comparison of infection intensity and prevalence of gastrointestinal parasites among categories of Dall’s sheep. (PDF)

S2 Table Models to test the association of gastrointestinal parasites and fitness indicators. (PDF)
