# Supplementary material for: Diversity of gastrointestinal helminths in Dall's sheep and the negative association of the abomasal nematode, Marshallagia marshalli, with fitness indicators
Source: PLoS One. 2018 Mar 14;13(3):e0192825. doi: 10.1371/journal.pone.0192825 (PMC5851548; doi:10.1371/journal.pone.0192825)
Supplement: S1 Table — (PDF) [file pone.0192825.s001.pdf]

S1 Table. Comparison of infection intensity and prevalence of gastrointestinal parasites among categories of Dall's sheep.

Infection Intensity by year (1971 v/s 1972)

-*Marshallagia marshalli*: Permutation test, Z = -0.6923, p-value = 0.4036
-*Moniezia* spp.: Permutation test,Z = 0.33765, p-value = 0.7976
-*Trichostrongylid* spp.: Permutation test, Z = -0.65415, p-value = 0.833
-*Trichuris schumakovitschi*: Permutation test, Z = -0.52134, p-value = 0.622
-Total Nematodirus spp.: Permutation test, Z = -0.72399, p-value = 0.4758
-Total intestinal worms: Permutation test, Z = 1.1078, p-value = 0.1415
-Total gastrointestinal worms: Permutation test, Z = -0.42376, p-value = 0.5698

Prevalence among years (1971 v/s 1972)

-*Marshallagia marshalli*: 100% of prevalence in both year categories
-*Moniezia* spp.: Monte Carlo X-squared = 2.4325, p-value = 0.2159
-*Trichostrongylid* spp.: Monte Carlo X-squared = 0.3322, p-value = 1
-*Trichuris schumakovitschi*: Monte Carlo X-squared = 1.7585, p-value = 0.2324
-Total Nematodirus spp.: 100% of prevalence in both year categories
-Total intestinal worms: 100% of prevalence in both year categories
-Total gastrointestinal worms: 100% of prevalence in both year categories

Infection intensity among yearlings and adults by species

-*Marshallagia marshalli*: t-test, t= 1.3717, df = 14.716, p-value = 0.1907
-*Moniezia* spp.: Permutation test, Z = 1.3872, p-value = 0.2197
-*Trichostrongylid* spp.: Permutation test, Z = 0.5119, p-value = 0.8712
-*Nematodirus archari*: Permutation test, Z = 1.2289, p-value = 0.2257
-*Nematodirus davtiani*: Permutation test, Z = 0.2852, p-value = 0.8882
-*Nematodirus oiratianus*: Permutation test, Z = -1.5405, p-value = 0.125
-*Nematodirus spathiger*: Permutation test, Z = 0.507, p-value = 0.6195
-*Trichuris schumakovitschi*: Permutation test, Z = -3.059, p-value = 0.0041
-*Skrjabinema ovis*: Permutation test, Z = -2.2791, p-value = 0.0256

-Total Nematodirus spp.: Permutation test, Z = -1.1304, p-value = 0.2556
-Total intestinal worms: Permutation test, Z = -1.2706, p-value = 0.8876
-Total gastrointestinal worms: Permutation test, Z = 0.6622, p-value = 0.286

Prevalence among yearlings and adults by species
-*Marshallagia marshalli*: 100% of prevalence in both age categories
-*Moniezia* spp.: Monte Carlo X-squared = 2.4325, p-value = 0.2159
-*Trichostrongylid* spp.: Monte Carlo X-squared = 0.3322, p-value = 1
-*Nematodirus archari*: Monte Carlo X-squared = 0, p-value = 1
-*Nematodirus davtiani*: Monte Carlo X-squared = 0.3005, p-value = 0.7141
-*Nematodirus oiratianus*: Monte Carlo X-squared = 1.4604, p-value = 0.2984
-*Nematodirus spathiger*: Monte Carlo X-squared = 0.4952, p-value = 1
-*Trichuris schumakovitschi*: Monte Carlo X-squared = 2.012, p-value = 0.2214
-*Skrjabinema ovis*: Monte Carlo X-squared = 4.0006, p-value = 0.08846
-Total Nematodirus spp.: Monte Carlo X-squared = 0.8568, p-value = 0.6202
-Total intestinal worms: 100% of prevalence in both age categories
-Total gastrointestinal worms: 100% of prevalence in both age categories

Intensity among pregnant and non-pregnant adult sheep by species
-*Marshallagia marshalli*: t-test, t= 2.4467, df = 24.807, p-value = 0.02185
-*Moniezia* spp.: Permutation test, Z = 1.3872, p-value = 0.2197
-*Trichostrongylid* spp.: Permutation test, Z = 0.5119, p-value = 0.8712
-*Nematodirus archari*: Permutation test, Z = 1.285, p-value = 0.2096
-*Nematodirus davtiani*: Permutation test, Z = 0.4917, p-value = 0.7886
-*Nematodirus oiratianus*: Permutation test, Z = 1.0285, p-value = 0.3414
-*Nematodirus spathiger*: Permutation test, Z = 1.221, p-value = 0.2281
-*Trichuris schumakovitschi*: Permutation test, Z = 1.7931, p-value = 0.069
-*Skrjabinema ovis*: Permutation test, Z = -0.2474, p-value = 0.8158
-Total intestinal worms: t-test, t = 0.959, df = 23.5, p-value = 0.3473

Intensity within species per age class
-*Marshallagia marshalli*: Permutation test, Z = 2.7157, p-value = 0.005601

Pairwise Comparison	W	p.value	p.adjust

1. Young adults - Yearlings =	-0.1375	0.8906	0.89060
2. Young adults - Mature adults =	2.681	0.00733	0.02199
3. Yearlings - Mature adults =	1.919	0.05494	0.08241

-*Moniezia* spp.: Permutation test, Z = 0.9547, p-value = 0.3735

-*Trichostrongylid* spp.: Permutation test, Z = -0.5, p-value = 0.7673

-*Nematodirus archari*: Permutation test, Z = 1.3859, p-value = 0.1659

-*Nematodirus davtiani*: Permutation test, Z = 0.407, p-value = 0.7047

-*Nematodirus oiratianus*: Permutation test, Z = -1.5182, p-value = 0.1301

-*Nematodirus spathiger*: Permutation test, Z = 0.9684, p-value = 0.3401

-*Trichuris schumakovitschi*: Permutation test, Z = -2.265, p-value = 0.0233

Pairwise Comparison	W	p.value	p.adjust
1. Young adults - Old adults =	0.6001	0.5484	0.69980
2. Young adults - Yearlings =	-3.081	0.00206	0.01236
3. Young adults - Mature adults =	-0.03297	0.9737	0.97370
4. Old adults - Yearlings =	-1.041	0.2977	0.59540
5. Old adults - Mature adults =	0.5488	0.5832	0.69980
6. Yearlings - Mature adults =	-2.851	0.004355	0.01306

-*Skrjabinema ovis*: Permutation test, Z = -1.4195, p-value = 0.1585

Intensity within species per body condition class

-*Marshallagia marshalli*: Permutation test, Z = -2.9659, p-value = 0.0013

Pairwise Comparison	W	p.value	p.adjust
1. Lean - Fair =	-1.26	0.2078	0.20780
2. Lean - Good =	-1.711	0.08703	0.14220
3. Lean - Very Good =	-2.674	0.007485	0.02491
4. Fair - Very Good =	-1.407	0.1593	0.19120
5. Fair - Good =	-1.671	0.09477	0.14220
6. Very Good - Good =	-1.673	0.09432	0.14220

-*Moniezia* spp.: Permutation test, Z = 0.5561, p-value = 0.6829

-*Trichostrongylid* spp.: Permutation test, Z = -0.4979, p-value = 0.7686

-*Nematodirus archari*: Permutation test, Z = -1.7482, p-value = 0.0784

-*Nematodirus davtiani*: Permutation test, Z = -1.4792, p-value = 0.1387

-*Nematodirus oiratianus*: Permutation test, Z = -1.4599, p-value = 0.1384

-*Nematodirus spathiger*: Categories with sample size lower than 5.

-*Trichuris schumakovitschi*: PermutationPermutation test, Z = -2.7731, p-value = 0.0049

Pairwise Comparison	W	p.value	p.adjust
1. Good - Very Good =	-1.456	0.1453	0.21800
2. Good - Fair =	-1.036	0.3002	0.35860
3. Good - Lean =	-1.821	0.06867	0.13730
4. Very Good - Fair =	-2.02	0.04337	0.13010
5. Very Good - Lean =	-2.816	0.004859	0.02915
6. Fair - Lean =	-0.918	0.3586	0.35860

-*Skrjabinema ovis*: Permutation test, Z = -0.47, p-value = 0.6525
