# Supplementary material for: Diversity of gastrointestinal helminths in Dall's sheep and the negative association of the abomasal nematode, Marshallagia marshalli, with fitness indicators
Source: PLoS One. 2018 Mar 14;13(3):e0192825. doi: 10.1371/journal.pone.0192825 (PMC5851548; doi:10.1371/journal.pone.0192825)
Supplement: S2 Table — (PDF) [file pone.0192825.s002.pdf]

S2 Table. Models to test the association of gastrointestinal parasites and fitness indicators.

	AIC	K	Deviance
**BCS**			
M. marshalli intensity^s, Intestine parasites intensity^s	99.5	3	89.7
M. marshalli intensity^s, Intestine parasites intensity^s, Pregnancy status	99.9	4	87.9
M. marshalli intensity^s, Intestine parasites intensity^s, Age	100.2	4	88.9
M. marshalli intensity, *T. schumakovitschi* intensity	101.6	3	91.6
M. marshalli intensity^s	101.8	2	93.8
M. marshalli intensity^s, Pregnancy status	102.1	3	92.1
M. marshalli intensity^s, Intestine parasites intensity, Pregnancy status, Intestine parasites intensity x Age	102.6	5	84.6
M. marshalli intensity^s, Pregnancy status, *M. marshalli* intensity x Pregnancy status	103.5	4	91.5
Pregnancy status^s, Intestine parasites intensity^s	152.8	3	142.9
Pregnancy status^s, Intestine parasites intensity^s, Age	157.6	4	141.6
Pregnancy status^s, Intestine parasites intensity^s, Age, Pregnancy status x Intestine parasites intensity	158.4	5	140.4
**SMI**			
M. marshalli intensity^s, *T. schumakovitschi* intensity	394.4	3	1347.2
M. marshalli intensity^s, Intestine parasites intensity	394.8	3	1356.8
M. marshalli intensity^s, Pregnancy status	395.1	3	1362.9
M. marshalli intensity^s, Pregnancy status, Intestine parasites intensity	396.6	4	1351.9
M. marshalli intensity^s, Pregnancy status, *M. marshalli* intensity x Pregnancy status	396.9	4	1358.4
M. marshalli intensity^s, Pregnancy status, Intestine parasites intensity, *M. marshalli* intensity x Pregnancy	398.5	5	1349.1
M. marshalli intensity^s, Pregnancy status, Age	400.7	4	1354.8
M. marshalli intensity^s, Pregnancy status, Intestine parasites intensity, Age	402.3	5	1346.4
M. marshalli intensity^s	464.6	2	1535.6
M. marshalli intensity^s, Intestine parasites intensity	466	3	1523.9
M. marshalli intensity^s, Intestine parasites intensity, Age	469.9	4	1448.6
Pregnancy status, Intestine parasites intensity, Age	521.3	4	1835.8
Pregnancy status^s	528.6	2	1955.1
Pregnancy status, Intestine parasites intensity	518.8	3	1912.3

Age	612.9	2	2062
(.)	614.4	1	2260.4
**PREGNANCY STATUS**			
M. marshalli intensity[s]	72.6	2	68.6
M. marshalli intensity[s], Age	74.0	3	68.0
M. marshalli intensity[s], Intestine parasite intensity	74.2	3	68.2
M. marshalli intensity, *T. schumakovitschi* intensity	74.2	3	68.2
M. marshalli intensity[s], SMI	74.5	3	68.5
M. marshalli intensity[s], Age	75.7	3	65.7
M. marshalli intensity[s], Age, *T. schumakovitschi* intensity	75.8	4	67.8
M. marshalli intensity, SMI, Intestine parasites intensity	76.1	4	68.1
M. marshalli intensity[s], SMI, Age	77.5	4	65.5
M. marshalli intensity, Age, Intestine parasites intensity	77.6	4	65.6
M. marshalli intensity, Age, Intestine parasites intensity, SMI	79.3	5	65.3
M. marshalli intensity x Age, Intestine parasites intensity, SMI	84	4	64

[s] Significant terms in the model
